# Eye Movement Alterations in Post-COVID-19 Condition: A Proof-of-Concept Study

**DOI:** 10.3390/s22041481

**Published:** 2022-02-14

**Authors:** Cecilia García Cena, Mariana Campos Costa, Roque Saltarén Pazmiño, Cristina Peixoto Santos, David Gómez-Andrés, Julián Benito-León

**Affiliations:** 1 ETSIDI-Center for Automation and Robotics, Universidad Politécnica de Madrid, Ronda de Valencia 3, 28012 Madrid, Spain; 2 CMEMS—UMinho, University of Minho, 4800-058 Guimaraes, Portugal; cristina@dei.uminho.pt; 3 LABBELS—Associate Laboratory, Braga, 4800-058 Guimaraes, Portugal; 4 ETSII-Center for Automation and Robotics, Universidad Politécnica de Madri, C/José Gutiérrez Abascal 2, 28006 Madrid, Spain; roquejacinto.saltaren@upm.es; 5 Paediatric Neurology, Vall d’Hebron University Hospital and VHIR (Euro-NMD, ERN-RND), 08035 Barcelona, Spain; david_gomez@vhebron.net; 6 Department of Neurology, University Hospital “12 de Octubre”, Av. de Córdoba, s/n, 28041 Madrid, Spain; jbenitol67@gmail.com; 7 Centro de Investigación Biomédica en Red sobre Enfermedades Neurodegenerativas (CIBERNED), 28031 Madrid, Spain; 8 Department of Medicine, Complutense University, Av. Séneca, 2, 28040 Madrid, Spain

**Keywords:** pathophysiology, eye movement, wearable gaze-tracker, post-COVID-19 condition, saccadic movement

## Abstract

There is much evidence pointing out eye movement alterations in several neurological diseases. To the best of our knowledge, this is the first video-oculography study describing potential alterations of eye movements in the post-COVID-19 condition. Visually guided saccades, memory-guided saccades, and antisaccades in horizontal axis were measured. In all visual tests, the stimulus was deployed with a gap condition. The duration of the test was between 5 and 7 min per participant. A group of n=9 patients with the post-COVID-19 condition was included in this study. Values were compared with a group (n=9) of healthy volunteers whom the SARS-CoV-2 virus had not infected. Features such as centripetal and centrifugal latencies, success rates in memory saccades, antisaccades, and blinks were computed. We found that patients with the post-COVID-19 condition had eye movement alterations mainly in centripetal latency in visually guided saccades, the success rate in memory-guided saccade test, latency in antisaccades, and its standard deviation, which suggests the involvement of frontoparietal networks. Further work is required to understand these eye movements’ alterations and their functional consequences.

## 1. Introduction

The measurement of eye movement and its alteration arises as a powerful marker to diagnose brain functionality. Several studies support this fact. For instance, in [1,2,3,4,5,6,7,8,9,10,11,12,13,14,15,16,17] provide scientific pieces of evidence related to particular alterations of eye movement in multiple neurological disorders, such as Alzheimer’s disease (AD), Parkinson’s disease (PD), multiple sclerosis (MS), and autism, among others. For example, patients with AD not only have higher latency and latency variability regardless of the tasks, but also show more incorrect antisaccades and take more time to correct them, which has been suggested as an early marker of AD [3,4,5,6,7]. In PD and some other parkinsonian syndromes, patients may present eye movement alterations, such as hypometria, abnormally fragmented saccades (multistep or staircase saccades), and considerable difficulty in inhibiting the saccade movement reflex during the antisaccade test. Furthermore, in patients with moderate or advanced PD, the latency in the visually guided saccades are higher compared with the controls [1,9].

In saccadic movements, several processes take place in the brain. For example, during the latency period, a shift of visual attention to the new target, a disengagement of oculomotor fixation, and a computation of the metrics of the movement are performed by the subject. These processes involve the activation of a large circuit that involves different cortical areas, including the parietal and the frontal lobes. Hence, the latency of eye movement is a cognitive–physiological parameter [11,15,16].

Coronavirus disease 2019 (COVID-19) primarily affects the respiratory system causing bilateral pneumonia, but it is increasingly being recognized as a systemic disease, with neurological manifestations reported even in patients with mild respiratory symptoms [18,19,20,21,22,23,24,25]. In fact, some patients with COVID-19 have clinical pictures similar to central nervous system infections, such as headache, epilepsy, and disturbed consciousness [24].

Neurotropism of SARS-CoV-2 infection has been established beyond doubt [18,19,20,21,22,23,24,25]. In this sense, many clinicians are expecting to see cases of post-COVID-19 neurological sequelae, mainly movement disorders [22] and other neurodegenerative diseases [26] in the upcoming decade.

There is little information about cognitive impairment in COVID-19 survivors. In a retrospective study in a large academic medical center in Chicago, Illinois, that included 50 hospitalized patients, 12 (24%) had cognitive abnormalities, particularly short-term memory impairment [27]. In a British cross-specialty surveillance study of acute neurological and psychiatric complications of COVID-19 that included 23 patients with neuropsychiatric disorders, six of them (26%) had a neurocognitive (dementia-like) syndrome [28]. There is also preliminary evidence of cognitive impairment after hospital discharge characterized by inattention, disorientation, and poorly organized movements in response to commands [29]. In a case series of four severe COVID-19 patients who required intensive care unit admission, cognitive impairment, identified as memory deficit and frontal syndrome, was detected after discharge [30].

We are now seeing that many COVID-19 survivors might have sustained postinfection sequelae, including fatigue, shortness of breath, and cognitive dysfunction, among others, which generally have an impact on everyday functioning [31]. These sequelae occur in patients with a history of probable or confirmed SARS-CoV-2 infection, usually three months from the onset, with symptoms that last for at least two months and cannot be explained by an alternative diagnosis. Known by a variety of names, including long COVID or long-haul COVID, are listed in the ICD-10 classification as post-COVID-19 condition [31].

Our aim is to study the potential eye movement alterations in post-COVID-19 patients. This assessment has the property of being non-invasive and cost-effective. Moreover, it is widely used to assess cognitive function in some neurological diseases, as previously mentioned. In this proof-of-concept, binocular eye movement was recorded using video-oculography. Images were processed under Matlab environments, and the most relevant features were computed and analyzed. To record the eye movement, we used a commercial and wearable gaze-tracker while a conventional chin rest immobilized the head of the participants. The visual stimulus was deployed in a conventional screen placed at 60 cm from the patient (the typical setup in all eye movements experiments) and its battery was programmed under a Python environment on an open-source platform called PsychoPy.

The ethical committees of the University Hospital 12 de Octubre, Madrid, Spain, approved this research.

The present article is organized as follows: Section 2 describes the Materials and Methods used in this proof-of-concept. Additionally, the full description of the eye movement tests, hardware, software, demographics, and clinical data of patients are deeply described in that section. Results are presented in Section 3, while the main findings are discussed in Section 4. Finally, conclusions and further clinical studies are summarized in Section 5.

## 2. Materials and Methods

A saccadic movement, or saccades, is defined as a rapid jerk-like movement of the eyes that direct the gaze to a new location and redeploy the region of high visual acuity centered on the fovea [32]. Saccades are regarded as voluntary movements, but are generally produced with highly automated routines. For further information related to the neurological aspects of eye movements, the reader is referred to [33].

### 2.1. Hardware and Software Description

**Figure 1:**
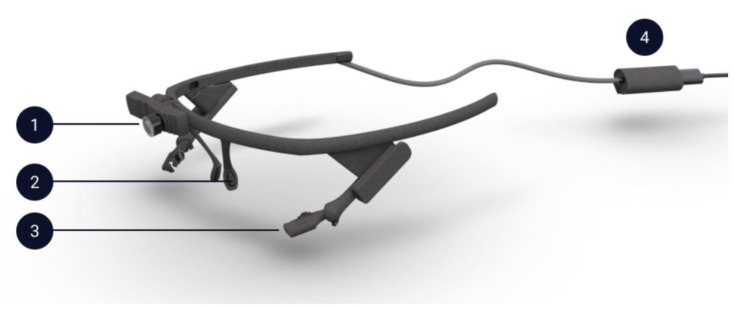
Pupil Core headset: (**1**) World Camera, (**2**) Nose Support, (**3**) Eye Cameras and (**4**) USB-C connector clip.

A commercial, binocular and wearable gaze tracker was used to record the eye movements. A picture of this gaze tracker model is shown in Figure 1 [34].

This whole system weighs around 22.75 g, and its dimensions are 160 mm × 51 mm × 175 mm. While the world camera is used to capture the image of the whole visual field of the user, each eye camera records the pupil position using infrared light. In this setup, the world camera operates at 30 Hz and each eye camera at 200 Hz with a resolution of 1080 × 1080 px and 192 × 192 px, respectively. According to user manual data, the gaze accuracy is 0.60º with a precision of 0.02º [35]. Moreover, the technical parameters reported by the manufactured turns it suitable for this proof-of-concept.

**Figure 2:**
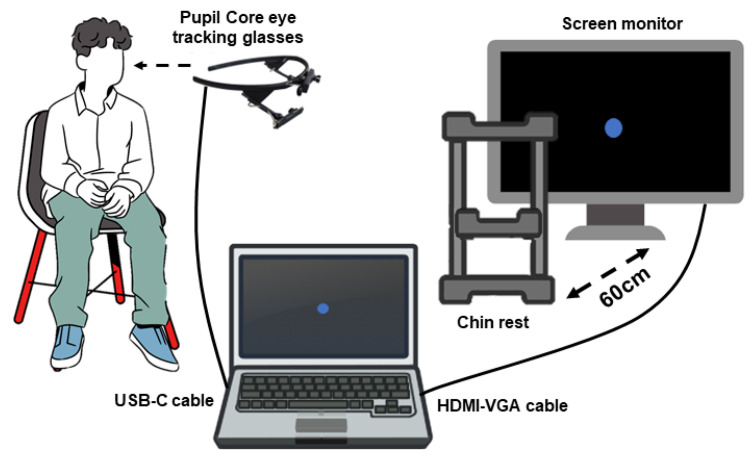
Scheme of the eye movements tests’ setup.

The subject wearing the gaze tracker was seated in front of the monitor with his/her head in the chin rest. We used a chin rest to keep the head still to avoid disturbance in the measurement of eye movements. The chin rest was placed at 60 cm from the monitor used to deploy the visual stimulus. The center of the monitor and the user’s line of sight were coincident as much as possible. The monitor was connected by an HDMI/VGA cable to a computer (ASUS VivoBook laptop CORE i7 8th Gen, in this specific case), while the Pupil Core eye-tracking glasses were also connected to the same laptop via USB-C cable. Figure 2 shows a scheme of the system setup. Notice that corrective glasses and make-up have to be removed.

The video-oculography system was calibrated before the recording using the calibration software provided by the manufacturer. It consisted of the onset of the stimulus in five different positions on the screen: the center and the four corners. The targets remained in each position for a few seconds while the pupil was automatically detected and that particular position of the gaze recorded.

In order to generate the battery of visual stimulus, it was used an open-source software called PsychoPy [36,37]. It is a free cross-platform package that allows running a wide range of experiments in the behavioral sciences (neuroscience, psychology, psychophysics, and linguistics, among others). It is a community project, so users have all the source code available to develop a new application. Moreover, it has a flexible and intuitive *Builder Interface* to develop a new experiment through a Python code or using the resources available in the application such as texts, icons, and figures, among others.

**Figure 3:**
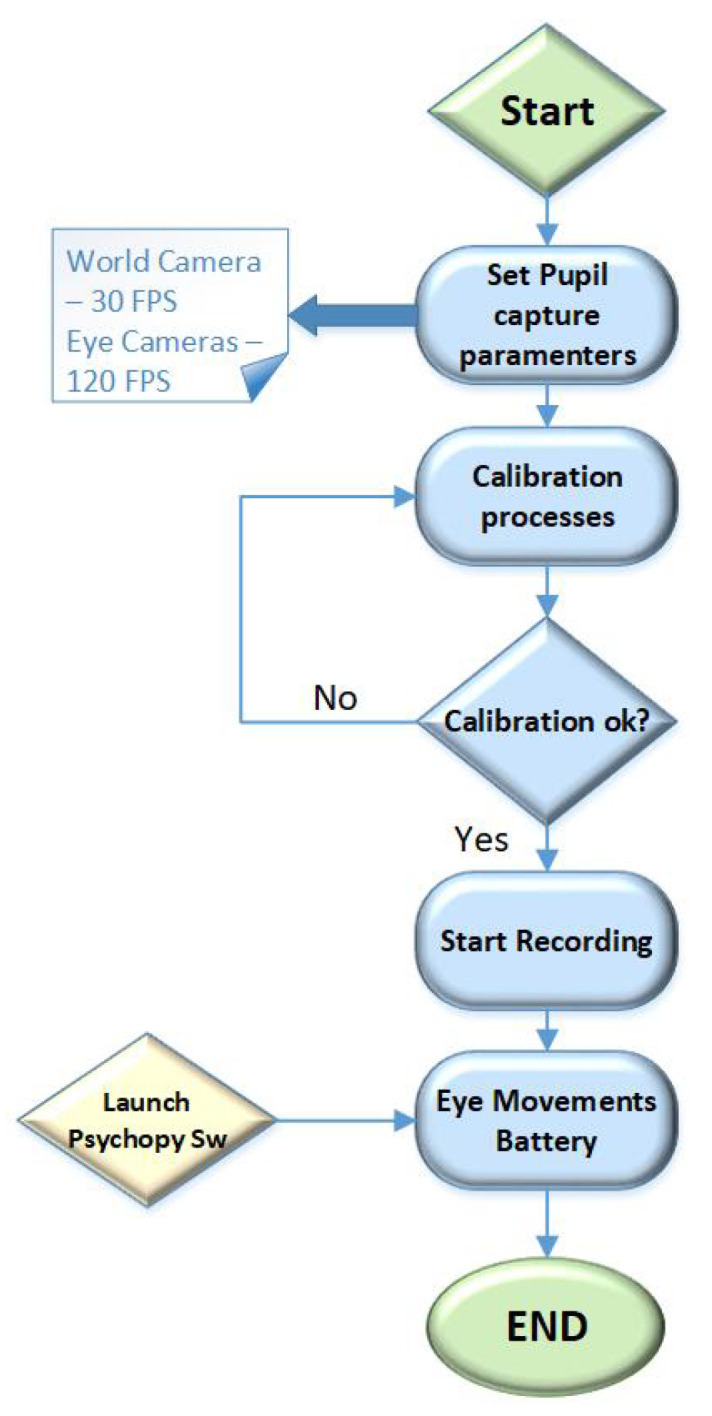
Eye movements test procedure.

It is worth emphasizing that Pupil software and *PsychoPy* software are manually synchronized, then the user is encouraged to stay quiet and not move the head after the calibration. Room lights were turned off to enhance the quality of the captured image by the gaze tracker. The procedure is outlined in Figure 3.

### 2.2. Description of the Eye Movement Battery

Based on previous studies [3,38,39,40,41,42] and as well as in authors expertise [2,16,43,44,45,46], in this proof of concept, it was decided to test the horizontal axis to validate the potential of the eye movement as a biomarker for the brain functionality in the post-COVID-19 condition.

In order to assess saccadic movements, there are three methods: the gap where the fixation point disappears prior to the onset of the visual stimulus; the shift where the offset of the fixation point and the onset of the visual stimulus happen simultaneously; and the overlap paradigms where the fixation point and the visual stimulus are overlapped in time before the offset of the former [47,48,49,50].

Saccadic latency is defined as the time (in milliseconds) from target appearance to saccade initiation of correct trials. Latency value is task dependant. In fact, there are several studies reporting these differences [5,17,51,52,53,54,55,56,57,58,59,60]. Perhaps, the most significant factor in reducing the reaction time, in terms of both the magnitude and robustness of the reduction, occurs in a phenomenon known as the *gap*. In a typical gap effect study, participants are asked to fixate on a centrally located fixation point and then make a saccade, as quickly as possible, to a suddenly appearing peripheral target. Several pieces of research justify the use of “gap” in attention and memory assessments such as.

This study implemented three visual tests under gap conditions: visually guided saccades, memory-guided saccades, and antisaccades. A calibration process was carried out before each test to ensure the accuracy of the measurement. As a stimulus, a blue circle with a diameter of 3 mm was selected, and the screen background was configured in dark black color. The stimulus location was $\pm 5^{\circ }$, $\pm 17^{\circ }$ and $\pm 22^{\circ }$. In all tests, the position of the stimulus (left or right, as well as the target amplitude) was randomized, and the gap duration was three seconds. In the following section, details related to each paradigm test are mentioned.

#### 2.2.1. Horizontal Visually Guided Saccade Test

**Figure 4:**
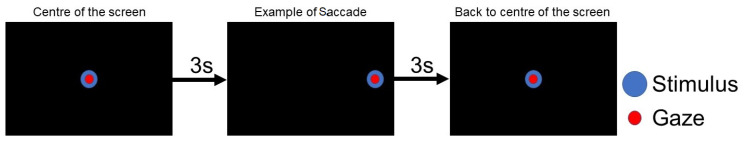
Horizontal visually guided saccade test.

As shown in Figure 4, the visual stimulus appears in the center of the screen (0,0) and jumps, randomly, towards a new position and, after three seconds, returns to the center. The whole duration of the test was one minute, and this process was repeated ten times. In this test, variables like latency towards the stimulus, latency back to the center of the screen, number of blinks, and performance were measured.

#### 2.2.2. Horizontal Memory-Guided Saccade Test

**Figure 5:**
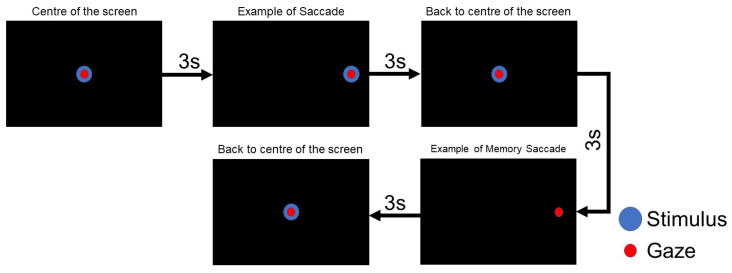
Horizontal memory guided saccade test.

In this test, the working memory [61,62] was assessed. We instructed to the subject to make a prosaccade during the first stimulus’s onset and then to remember this position in order to do a memory saccade in the dark period. Figure 5 summarizes this procedure: the visual stimulus appears in the center of the screen, after 3 s, the stimulus jumps randomly to right or left, with unpredictable eccentricity, $\pm 5^{\circ }$, $\pm 17^{\circ }$ and $\pm 22^{\circ }$ and returns to the screen’s center. After that, the participant must remember the last position of the visual stimulus and generate a voluntary eye movement towards it, but in the absence of a stimulus. The whole duration of the test is two minutes, and variables such as blinks and success rate in the memory saccade are computed.

#### 2.2.3. Horizontal Visually Antisaccade Test

**Figure 6:**
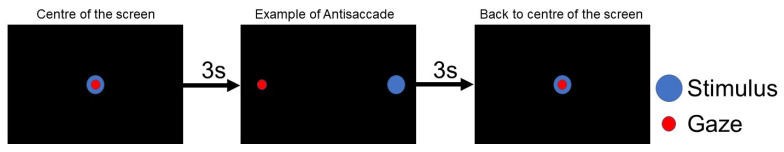
Antisaccade test scheme.

Similar to the horizontal visually guided saccade test, in the antisaccade test, the stimulus appears first in the center and then in random positions for three seconds in both cases. However, the participant is encouraged to look at the opposite position of the visual stimulus. This process is repeated ten times, and the whole duration of the test is one minute. In Figure 6, a scheme of antisaccade test is presented. The performance of the antisaccade test requires at least two subprocesses: the ability to suppress a reflexive saccade towards the visual stimulus and the ability to generate a voluntary saccade in the opposite direction to a location without any stimulus. Blinks, success in the antisaccades, the latency of the antisaccades and reflexive antisaccades were calculated.

#### 2.2.4. Eye Movement Features Characterization

**Figure 7:**
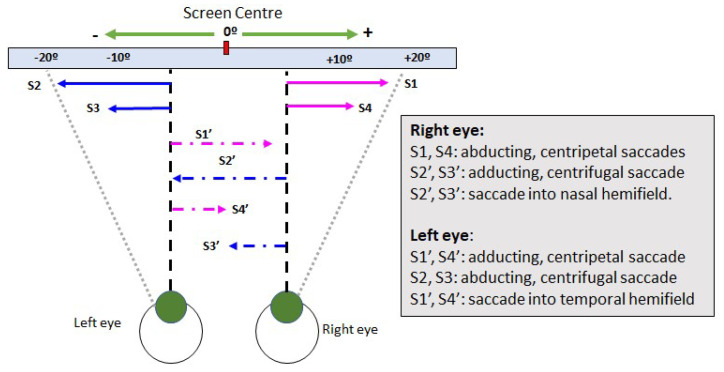
Binocular saccadic movements scheme.

This subsection defines the features computed in eye movements tests described in the previous subsection. After recording, each test is processed using the gaze-tracker software that provides binocular gaze positions in both axes, x, and y. Figure 7 shows a scheme with the saccadic movements for each eye. Here, the movement is classified according to the movement itself (adducting or abducting), the visual pathway (temporal or nasal), and the primary position of the eyeball (centrifugal and centripetal). *Centripetal saccades* are made from the periphery of the orbit to its center (the primary position of the eyeball), while *centrifugal saccades* are made from the center to the periphery. These saccadic movements involve the adduction and the abduction of the eyeball.

**Figure 8:**
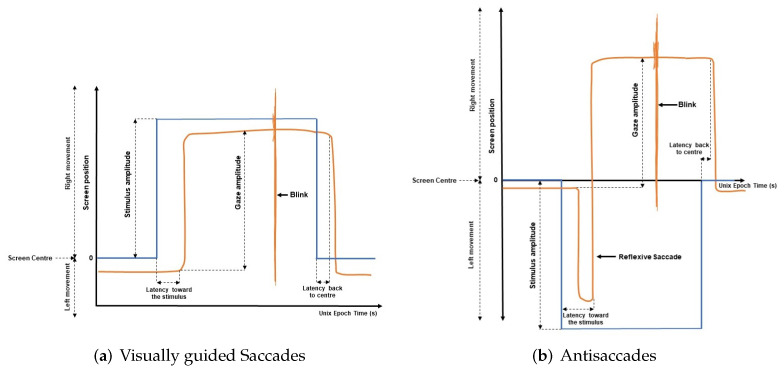
Gap paradigm. Features computed in the eye movement tests.

Figure 8 shows the features considered in this research. Blinks are automatically detected and exported by the gaze tracker software.

Centrifugal latency, ▵t [ms], is defined by the time elapsed between the onset of the periphery stimulus, $t_{s}$, and the time from the eye moves in response to this stimulus $t_{gaze}$. It is computed by (1);

(1) $$▵t=t_{gaze}-t_{s}$$

Centripetal Latency, $▵t_{c}$ [ms], is defined by the time elapsed between the onset of the fixation point in the center of the screen, $t_{fixpoint}$, and the time from the eye moves in response to it, $t_{gaze}$. In this case, it is computed by (2);

(2) $$▵t_{c}=t_{gaze}-t_{fixpoint}$$

In [44] was demonstrated the linear relation between age and the ▵t and $▵t_{c}$ for healthy volunteers, and a model was provided for computing the latency under gap paradigm. That research used the centrifugal and centripetal latencies model to compare the measured values to ensure the measured value’s reliability. In (3) and (4), the linear dependency between latency and age in healthy brain aging was demonstrated.

(3) $$▵t_{model}\left[ms\right]=187.16+0.55*age$$

(4) $$▵t_{cmodel}\left[ms\right]=162.22+0.89*age$$

According to [16], *Reflexive Saccade* is defined by an eye movement in the same direction of the stimulus, in the antisaccade test (see Figure 8b). The *Success Rate* in antisaccade test or in memory guided test is defined by the correct movement according to the test (opposite side in antisaccade and remember position in memory test, respectively).

When the centripetal latency, $▵t_{c}$, is between 70 ms and 130 ms, the eye movement is usually referred to as *express saccade* [63] and is driven by the subcortical retinotectal pathways [64]. In contrast, the “normal” saccades are driven by the thalamocortical pathway, which projects to the parietal eye field and then to the superior colliculus [65], so express saccade is not included in mean values.

This study did not include measurements of dysmetrias and related variables and asymmetries in the movement (differences between eyes).

### 2.3. Healthy Controls and Patients with the Post-COVID-19 Condition

A group of ten patients with the post-COVID-19 condition was consecutively selected from the *Patient Affected by COVID-19 Disease Association* (AMACOVID), Madrid. None reported having a neurological disease previous to COVID-19. One of the subjects was excluded after the data analysis due to noise in the measurement; therefore, nine participants (three men and six women) were included in the analyses. The patients with the COVID-19 condition had a mean (±standard deviation) age of 49.56 ± 9.14 years, weight of 74.33 ± 19.29 kg, and height of 164.0 ± 7.27 cm. On the other hand, a group of healthy volunteers matched by age was recruited as a control group. These subjects had not been diagnosed with COVID-19 disease nor had previous neurological diseases [44]. See Table 1 for details.

Table 2 shows the most relevant demographic and clinical characteristics of post-COVID-19 patients.

Table 3 summarizes the clinical picture suffered by the patients upon the diagnosis, including the diagnosis date. COVID-19 diagnosis was confirmed by SARS-CoV-2 reverse transcription-polymerase chain reaction of a nasopharyngeal swab. Three patients were infected during the first wave (February to 1 June 2020), four were infected during the second wave (2 June 2020–9 December 2020), and the remaining two during the third wave (10 December 2020–15 March 2021). The most common clinical picture was febrile illness followed by cough and pneumonia. Patients’ other symptoms were hair and skin problems, headache, anorexia, and diarrhea, among others.

Table 4 summarizes the symptoms suffered by patients after COVID-19. The majority of them present complaints of fatigue or memory disturbance. Other symptoms reported were anosmia and shortness of breath. Patients VAMA02 and VAMA03 mentioned abnormal heart rhythms, such as arrhythmias and tachycardia, respectively. On the other hand, VAMA03 had edemas of the lower limbs and VAMA10 skin rash; hair loss was reported in both patients. This last patient also complained of hand pain and VAMA06 that had limb weakness. In addition, VAMA03 had speech problems and hand pain. VAMA06 had limb weakness as well.

## 3. Results

### 3.1. Validation of the Measurement of the Group of Healthy Volunteers

Table 5, Table 6 and Table 7 present the computed eye movement’s features described in previous section. Table 5 shows the minimum, maximum, and mean value of the latency, as well as the value computed by the model given by Equations (3) and (4). By computing the absolute error in each measurement, we found that this error ranged between $8\times 10^{-2}$ and $6\times 10^{-3}$ ms; hence, the values measured with the portable gaze tracker had the accuracy needed to compute latency.

Average antisaccade error rates in healthy humans vary considerably across studies and laboratories, with some studies reporting rates as low as 5% and others as high as 25% [66,67]. Some studies [68,69] using large samples suggest an error rate of around 20% is typical. Error rates are not constant across the lifespan, being highest during childhood, reaching the lower rates during early adulthood, and then increasing very slowly with advancing age until around 55–60, when the rate of increase appears to accelerate [44].

The latency values depend on the target location tested as well as the method to assess it (gap, step or overlap) [70]. Several studies report the values of latency in visually guided saccade test with a large number of volunteers [71,72,73,74]. By comparing results given in Table 7 with those previously reported, we found similar latency values. According to memory-guided saccades and antisaccades tests, parameters measured from group G1 were in the range of those previous studies related to the success of the ocular tasks [44,75,76]. The main conclusion here is that the gaze-tracker is helpful for this proof of concept.

### 3.2. Post-COVID-19 Patients Measurement

By considering the eye movement battery described in the previous section, the main results are presented in Table 8, Table 9 and Table 10. According to this, and by comparing them with those previously studies [18,77,78], these patients had eye movement alterations.

Two patients had latencies out of the normal range for their age (VAMA02 and VAMA10). Considering the success rate in the memory-guided saccade test, three patients showed abnormal success rates (VAMA05, VAMA06, and VAMA10), less than 50%. In the antisaccade test, the total number of correct eye movements (opposite to the stimulus) were according to the normality; however, three patients had a poor performance in the inhibitory capabilities (VAMA02, VAMA06, and VAMA10). Furthermore, the latency of the antisaccades was proper of healthy volunteers in the range of 65–85 years old [44]. The number of blinks was also higher than healthy volunteers in all tests.

### 3.3. Statistical Analysis

Although the sample size in both groups was small, we checked the statistical significance of the features. Firstly, variables were classified as parametric or non-parametric. Most part of the values shown in Table 5, Table 6, Table 7, Table 8, Table 9 and Table 10 are expressed as the mean value and the standard deviation. Differences between groups were analyzed using t-test for parametric variables while Kruskal–Wallis test followed by Dunn’s test for non-parametric variables. This analysis was performed using MatLab.

There were significant differences among groups when *p*-value was less than 0.05. After this, significative variables were: centripetal latency in visually guided saccades (*p*-value < 0.05), the success rate in memory-guided saccade test had a *p*-value < 0.05, blinks in whole test (*p*-value < 0.02), latency in antisaccades (*p*-value < 0.001) and its standard deviation (*p*-value <0.001). Although the p-value for the centrifugal latency was not significant (>0.05), it could be due to the small sample size.

## 4. Discussion

In this study, we have reported the eye movement alterations in a group of post-COVID-19 patients. Despite the gaze tracker was not certified as a medical device, the computed values from the gaze position given by the gaze tracker were in agreement with the literature (see Section 3). Therefore, the gaze-tracker and the software developed to assess the main features are valid.

According to [70], using monocular recordings of eye movement in monocular viewing conditions or binocular recordings in binocular viewing conditions leads to asymmetries in the retina and the optic nerve, which affects saccadic eye movements, meanly in peak velocity than other parameters [79]. This study measured binocular eye movements and compared results with other studies done in binocular viewing conditions.

The values measured reveal that patients who have suffered COVID-19 have eye movement alterations of interest, as these alterations are similar to those reported for mild cognitive impairment or Alzheimer’s disease [3,4,5,6,7,9,11,12,15,16].

The dorsolateral prefrontal cortex is involved in the development of saccadic eye movements [80]. This structure has direct connections with the main cortical ocular motor areas, namely the frontal eye field and the supplementary eye field in the frontal lobe; several (associative, attentional, and motor) areas in the posterior parietal cortex, including the parietal eye field; the cingulate eye field in the anterior cingulate cortex; and the superior colliculus in the brainstem [80]. For the performance of memory-guided saccades, that is, involving short-term spatial memory, visually guided saccades, and antisaccades, the posterior parietal cortex, the dorsolateral prefrontal cortex, and the frontal eye field play significant roles [80,81,82,83]. We found that patients with the post-COVID-19 condition had eye movement alterations mainly in centripetal latency in visually guided saccades, the success rate in memory-guided saccade test, latency in antisaccades, and its standard deviation, which suggests the involvement of frontoparietal networks. Further work is required to understand these eye movements’ alterations and their functional consequences.

The main limitations of our study are the sample size and the age of volunteers. We, however, considered that the sample size was enough for a proof-of-concept study. Likewise, related to the second limitation, the age as well as the sex of the participants were not relevant to demonstrating that SARS-CoV-2 virus infection may affect eye movement performance.

## 5. Conclusions

This study aimed to evaluate eye movement alterations using wearable and a commercial gaze-tracker.

We used a chin rest to avoid the influence of head movements on eye movements. In addition, after each visual test, the system was re-calibrated. Furthermore, those ocular features highly dependent on the accuracy were not included, such as gain or dysmetria.

Related to the visual tests, we implemented them using a free software platform under Python. The gap setup was selected because it is widely used to assess memory conditions in other neurological diseases like mild cognitive impairment, AD, or PD, (see Section 1 and Section 2).

Our patients still report cognitive complaints that they did not have before COVID-19. Eye movement assessment objectively unveiled the existence of these symptoms. Ongoing, more extensive studies will better understand the impact of COVID-19 on the brain.

## Figures and Tables

**Table 1:** Demographic and clinical characteristics of healthy controls.

ID	Age (Range)	Sex	Cardiovascular Diseases	Diabetes Mellitus
H1	51–60	Female	Arterial hypertension	No
H2	<19	Male	Arrhythmia	No
H3	<19	Male	None	No
H4	20–40	Male	None	No
H5	20–40	Male	None	No
H6	20–40	Male	None	No
H7	20–40	Female	None	No
H8	20–40	Male	None	No
H9	41–50	Male	None	No

**Table 2:** Demographic, clinical characteristics and medical history of patients before COVID-19 diagnosis.

Patient	Age	Sex	Laboral Activity	Cardiovascular Diseases	Diabetes Mellitus
VAMA01	59	Female	Association coordinator	None	Yes
VAMA02	54	Male	Commercial tasks	Supraventricular arrythmia (14y)	No
VAMA03	28	Female	Housewife	None	No
VAMA04	44	Female	Housewife	None	No
VAMA05	60	Male	Caretaker and maintenance in a school	None	No
VAMA06	49	Female	Cleaner	None	No
VAMA08	46	Female	Call center	None	No
VAMA09	54	Male	Worker	None	No
VAMA10	52	Female	Call center	None	No

**Table 3:** Clinical picture upon diagnosis.

Patient	Diagnosis Date (mm/dd/yyyy)	Diarrhea	Pneumonia	Fever	Cough	Headache	Gastrointestinal Symptoms
VAMA01	04/04/2020	X	X	X	-	-	-
VAMA02	03/03/2020	-	X	X	X	-	-
VAMA03	08/28/2020	X	-	X	-	X	X
VAMA04	12/17/2020	-	X	X	X	X	Anorexia
VAMA05	09/03/2020	-	X (bilateral)	X	X	-	Anorexia
VAMA06	12/29/2020	-	-	X	X	-	-
VAMA08	12/29/2020	-	-	X	-	X	-
VAMA09	12/02/2020	-	Mild	Low-grade	-	-	-
VAMA10	03/15/2020	X	-	-	X	X	X

**Table 4:** Post-COVID-19 symptoms.

Patient	Anosmia	Memory Complaints	Fatigue	Shortness of Breath
VAMA01	X	X	X	-
VAMA02	-	-	X	X
VAMA03	-	X	-	X
VAMA04	X	X	X	-
VAMA05	-	-	X	X (on walking)
VAMA06	-	X	X	-
VAMA08	X	X	X	-
VAMA09	-	X	X	X
VAMA10	-	X	X	X (feeling of chest pressure)

**Table 5:** Visually guided saccade test: latencies values for the control group.

ID	Blinks	▵t [s] $▵t_{\min}$–$▵t_{\max}$	$▵t_{\mathrm{mean}}$± SD	$▵t_{\mathrm{model}}$	$▵t_{c\mathrm{mean}}$± SD	$▵t_{c\mathrm{model}}$
H1	0	0.198–0.272	0.223 ± 0.02	0.219	0.213 ± 0.02	0.214
H2	3	0.132–0.451	0.212 ± 0.09	0.197	0.148 ± 0.02	0.178
H3	0	0.151–0.383	0.212 ± 0.06	0.197	0.202 ± 0.06	0.178
H4	4	0.156–0.280	0.198 ± 0.03	0.200	0.216 ± 0.03	0.183
H5	0	0.154–0.267	0.192 ± 0.03	0.202	0.189 ± 0.03	0.184
H6	2	0.132–0.239	0.181 ± 0.03	0.199	0.162 ± 0.05	0.180
H7	8	0.151–0.269	0.180 ± 0.03	0.201	0.203 ± 0.07	0.185
H8	0	0.137–0.233	0.176 ± 0.03	0.203	0.172 ± 0.02	0.188
H9	3	0.214–0.338	0.297 ± 0.06	0.211	0.208 ± 0.02	0.200
Mean Values	2.2	0.158–0.303	0.209 ± 0.04	0.203	0.190 ± 0.03	0.187

**Table 6:** Memory-guided saccade test: success rate in memory saccade for healthy control group.

ID	Blinks	Success Rate
H1	3	100%
H2	12	100%
H3	0	100%
H4	2	100%
H5	0	90%
H6	2	100%
H7	21	90%
H8	4	100%
H9	3	100%
Mean Values	5.2	97.77%

**Table 7:** Antisaccadic test: main features for the healthy control group.

ID	Blinks	Correct	Reflexive	Success Rate	Latency [s] Min–Max	Mean ± SD
H1	2	100%	37.5%	62.5%	0.160–0.659	0.316 ± 0.135
H2	8	100%	50%	50%	0.197–0.511	0.330 ± 0.092
H3	0	100%	60%	40%	0.219–0.612	0.336 ± 0.103
H4	0	100%	60%	40%	0.210–0.463	0.317 ± 0.078
H5	1	80%	40%	40%	0.216–0.679	0.396 ± 0.135
H6	3	100%	0%	100%	0.162–0.449	0.232 ± 0.079
H7	0	70%	40%	30%	0.122–0.672	0.368 ± 0.140
H8	0	100%	60%	60%	0.180–0.469	0.273 ± 0.092
H9	1	100%	50%	50%	0.197–0.398	0.315 ± 0.044
Mean Values	1.6	94.4%	44.2%	52.5%	0.184–0.545	0.320 ± 0.099

**Table 8:** Visually guided saccade test: latencies values in post-COVID-19 patients.

Patient	Blinks	▵t [s] $▵t_{\min}$–$▵t_{\max}$	$▵t_{\mathrm{mean}}$± SD	$▵t_{c\mathrm{mean}}$± SD
VAMA01	2	0.162–0.327	0.216 ± 0.04	0.200 ± 0.02
VAMA02	0	0.168–0.741	0.264 ± 0.16	0.255 ± 0.07
VAMA03	0	0.177–0.361	0.248 ± 0.05	0.223 ± 0.03
VAMA04	9	0.182–0.342	0.233 ± 0.05	0.214 ± 0.05
VAMA05	6	0.168–0.330	0.234 ± 0.05	0.212 ± 0.05
VAMA06	54	0.170–0.274	0.210 ± 0.03	0.213 ± 0.01
VAMA08	11	0.167–0.302	0.226 ± 0.04	0.214± 0.04
VAMA09	22	0.164–0.255	0.216 ± 0.03	0.223 ± 0.03
VAMA10	3	0.214–0.712	0.496 ± 0.16	0.433 ± 0.15
Mean Values	11.8	0.174–0.404	0.260 ± 0.07	0.243 ± 0.05

**Table 9:** Memory-guided saccade test: success rate in post-COVID-19 patients.

Patient	Blinks	Success Rate
VAMA01	18	100%
VAMA02	0	80%
VAMA03	2	100%
VAMA04	18	100%
VAMA05	22	40%
VAMA06	53	0%
VAMA08	56	100%
VAMA09	20	100%
VAMA10	7	20%
Mean Values	21.7	71.1%

**Table 10:** Antisaccadic test. Main features in post-COVID-19 patients.

Patient	Blinks	Correct	Reflexive	Success Rate	Latency [sec] Min–Max	Mean ± SD
VAMA01	9	100%	10%	90%	0.227–0.630	0.438 ± 0.112
VAMA02	1	60%	100%	0%	0.177–0.737	0.472 ± 0.184
VAMA03	0	100%	90%	10%	0.272–0.934	0.417 ± 0.149
VAMA04	5	100%	60%	40%	0.240–0.831	0.432 ± 0.137
VAMA05	4	100%	40%	60%	0.219–0.860	0.333 ± 0.157
VAMA06	34	70%	40%	30%	0.227–1.499	0.461 ± 0.326
VAMA08	26	90%	30%	60%	0.257–0.574	0.358 ± 0.081
VAMA09	27	90%	70%	20%	0.246–0.888	0.468 ± 0.182
VAMA10	7	80%	60%	20%	0.275–0.948	0.499 ± 0.183
Mean Values	12.5	87.8%	55.5%	36.6%	0.237–0.878	0.430 ± 0.167

## Data Availability

The data sets generated during the current study are available from the corresponding author upon reasonable request.
